# A Three-Year Retrospective Study Assessing the Quality of the Course of Management of Infective Endocarditis in a Tertiary Hospital in Riyadh, Saudi Arabia

**DOI:** 10.7759/cureus.37539

**Published:** 2023-04-13

**Authors:** Nejood Alsheikh, Ghadah B Alghbewi, Noof A Hakami, Soad Aljudaie, Fatmah Sohaibani, Mohammed Alsaif, Majid Alsalamah

**Affiliations:** 1 College of Medicine, King Saud Bin Abdulaziz University for Health Sciences, Riyadh, SAU; 2 Emergency Department, Ministry of the National Guard - Health Affairs, Riyadh, SAU; 3 Emergency Department, King Saud Bin Abdulaziz University for Health Sciences, Riyadh, SAU

**Keywords:** antibiotic, echocardiography, empirical antibiotic therapy, infective endocarditis, endocarditis

## Abstract

Background: Infective endocarditis (IE) is one of the most misdiagnosed diseases in Saudi Arabia because of the variable treatment regimen. This study aims to assess the quality of the management of infective endocarditis in a tertiary care teaching hospital.

Methods: A single-center retrospective cohort study was conducted, based on electronic medical records extracted from the BestCare electronic medical record system, of all patients who presented with infective endocarditis as a final diagnosis from 2016 to 2019.

Results: Out of a total of 99 patients diagnosed with infective endocarditis, 75% of our patients had blood cultures ordered before initiating empirical antibiotic therapy. Positive blood cultures were reported in 60% of patients. *Staphylococcus aureus* was the most common organism, identified in 18% of our patients, followed by *Streptococcus viridans* at 5%. Empirical antibiotics were initiated in 81% of patients. Proper antibiotic coverage was initiated within a week for 53% of the patients, and 14% had proper antibiotic coverage within two weeks. On echocardiography, 62% of the patients had vegetation that was present in a single valve. The mitral valve had the highest incidence of vegetation (24%), followed by the aortic valve (21%). Follow-up echocardiography was done in 52% of patients. It showed regressed vegetation in 43% of patients, while only 9% of patients had no vegetation regression. Valve repair was done in 25% of patients. Out of 99 patients, 47 required ICU admission. The mortality rate was 18%.

Conclusion: Overall management of infective endocarditis in the study hospital was appropriate and highly compliant with guidelines, with a few areas that could be improved further.

## Introduction

Infective endocarditis (IE) is a bacterial infection that affects the innermost surface of the heart, the endocardium, and typically affects the heart valves [1]. In the US, IE affects 15 out of every 100,000 people each year, and the number continues to rise [2]. We did a literature review and found one study in Saudi Arabia that was conducted between January 1995 and December 2008. The study consisted of 83 cases of IE. They found that out of the 83 cases, only 54 patients were diagnosed with definitive IE, and the remaining were potential IE cases [3]. This leads to the central issue related to IE, which is the diagnostic challenges and treatment dilemmas associated with it. This is mainly due to the bacterial causative species advancing with time, a change in the risk factors and patient demographics, as well as the irregular case reports of unusual and uncommon causative organisms. Therefore, even with the development of new diagnostic methods such as polymerase chain reaction to help assess all types of cases, the evolution of the typical IE bacteria makes it difficult to diagnose [4].

Moreover, the variable clinical manifestations complicate the recognition of IE cases, where early, effective antibiotics or cardiac valve surgery would benefit the patients. Excluding IE is crucial, not only to avoid the administration of unnecessary antibiotics but also to focus on other diagnosis possibilities. Even with the introduction and usage of new diagnostic methods, there was no remarkable evidence stating that these techniques could guide or aid physicians in choosing regimens of antimicrobial drugs that should be used for treatment in the case of an identified organism. Another issue that is being faced is the hindrance of culturing endocarditis-causing Gram-negative organisms to test for susceptibility, which leaves it harder for physicians to determine which regimen is most appropriate [5].

For the reasons mentioned above, it was found that infective endocarditis is one of the diseases that is often subjected to medical errors [6-8]. Therefore, this study aims to assess the quality of the management of infective endocarditis in a teaching hospital.

## Materials and methods

Study design and setting

This study was a three-year descriptive cross-sectional retrospective study based on data extracted from an electronic hospital information system (BESTcare) of patients presenting at a tertiary teaching medical center in Riyadh, Saudi Arabia, with infective endocarditis as a final diagnosis.

Study participants

The study included all adult and pediatric patients admitted or presented to the ER from January 1, 2016 to December 31, 2019, with a confirmed diagnosis of infective endocarditis. The study excluded patients with misdiagnosed infective endocarditis.

Data analysis 

The data included demographic information, symptoms, comorbidities, culture results, management, echo findings, and patient outcome. A descriptive analysis based on frequency and percent distribution was done for all variables.

Ethical considerations

A database used for data collection was secured for patients' confidentiality under the supervision of the primary investigator. An institutional review board (IRB) approval was obtained on August 24, 2020, from King Abdullah International Medical Research Center, Riyadh, Saudi Arabia (Memo Ref. No. IRBC/1439/20). No conflict of interest was present. Furthermore, no consent was needed from the patients since this study is a retrospective one. The patients’ information was secured and accessible only by the co-investigators and primary investigator. Each patient’s medical record number was assigned a certain serial number to further protect their information. All medical records were kept on password-protected computers to ensure the hidden identities of all patients. This study abided by the principles of the Declaration of Helsinki.

## Results

Ninety-nine patients of all ages presenting with infective endocarditis between 2016 and 2019 were included in our study. Table 1 shows the baseline characteristics of the population. Out of 99 patients, 68 (68%) were men and the rest were women (32%). All age groups had similar incidence rates, with the age group 41-65 years having the highest incidence (n = 28, 28%), and the age group 0-18 years having the lowest incidence (n = 19, 19%). Ninety-five percent of the patients had a previous chronic disease; hypertension had the highest incidence (n = 39, 39%), followed by diabetes (n = 37, 37%). Out of 99 patients, 41 (41%) had a previous cardiac pathology, and 19 (19%) had previous procedures. Fever was the most frequent symptom (n = 62, 63%), followed by shortness of breath (n = 23, 23%), and fatigue (n = 12, 12%).

**Table 1:** Baseline characteristics. ESRD: end-stage renal disease, SLE: systemic lupus erythematosus, CABG: coronary artery bypass grafting, TGA: transposition of the great arteries, PFO: patent foramen ovale, PCI: percutaneous coronary intervention, VSD: ventricular septal defect, ASD: atrial septal defect

Variables		n (N=99)	%
Age	0–18	19	19%
	19–40	25	25%
	41–65	28	28%
	Above 65	27	27%
Gender	Male	67	68%
	Female	32	32%
Past medical history	Hypertension	39	39%
	Diabetes	37	37%
	Prosthetic valve	21	21%
	Rheumatic heart disease	14	14%
	ESRD	11	11%
	Heart failure	9	9%
	Previous I.E.	7	7%
	Chronic kidney disease	4	4%
	History of brucellosis	3	3%
	Down’s syndrome	2	2%
	SLE	2	2%
	Drug abuse	1	1%
Previous cardiac pathology	VSD	10	10%
	Mitral regurgitation	4	4%
	Aortic stenosis	3	3%
	Non-dilated cardiomyopathy	3	3%
	ASD	3	3%
	Aortic regurgitation	2	2%
	Mitral stenosis	2	2%
	Tetralogy of Fallot	2	2%
	Dilated cardiomyopathy	2	2%
	PDA	2	2%
	Tricuspid regurgitation	1	1%
	Bicuspid aortic valve	1	1%
	TGA	1	1%
	Aortic atresia	1	1%
	Interrupted aortic arch	1	1%
	PFO	1	1%
	Sinus of Valsalva ruptures	1	1%
	Coronary cusp prolapse	1	1%
Dysrhythmias	Atrial fibrillation	6	6%
	Atrial Flutter	1	1%
	Type 2 AV block	1	1%
Previous procedures	CABG	9	9%
	Repair/replacement of valve	6	6%
	Pacemaker	2	2%
	PCI	1	1%
	ICD	1	1%
Most common chief complaints	Fever	62	62%
	Shortness of breath	23	23%
	Fatigue	12	12%
	Chills and rigors	12	12%
	Vomiting	8	8%

We looked at the blood culture results of all the included patients. The blood culture results are shown in Table 2. Among the 99 patients, 74 (75%) had a blood culture ordered before initiating empirical antibiotic therapy. A positive blood culture was reported in 60 patients (61%), where 21 different organisms were identified. *Staphylococcus aureus* had the highest incidence (n = 18, 18%). Clindamycin was the most susceptible antibiotic (n =18, 18%).

**Table 2:** The blood culture results of all patients diagnosed with infective endocarditis.

Variables		n (N=99)	%
Frequency of blood cultures drawn prior to any antibiotic treatment		74	75%
Results of all the blood cultures	Positive	60	60%
	Negative	31	31%
	Scanty growth	8	8%
Organisms identified by the blood cultures	Staphylococcus aureus	18	18%
	Streptococcus viridans	5	5%
	Enterococcus faecalis	5	5%
	Methicillin-resistant *S. aureus*	5	5%
	Staphylococcus epidermidis	5	5%
	Pseudomonas aeruginosa	3	3%
	Staphylococcus hominis	3	3%
	Brucella sp	2	2%
	Abiotrophia defectiva	2	2%
	Streptococcus salivarius	1	1%
	Staphylococcus warneri	1	1%
	Streptococcus gordonii	1	1%
	Streptococcus gallolyticus	1	1%
	*Streptococcus pyogenes* A	1	1%
	Corynebacterium striatum	1	1%
	Streptococcus mitis	1	1%
	Ralstonia mannitolilytica	1	1%
	Staphylococcus haemolyticus	1	1%
	Agalactia	1	1%
	Coagulase-negative staph	1	1%
	Gram-negative bacteria	1	1%
Antibiotics found to be susceptible	Clindamycin	18	18%
	Oxacillin	17	17%
	Cefazolin	17	17%
	Vancomycin	16	16%
	Gentamicin	14	14%
	Ampicillin	7	7%
	Cefotaxime	5	5%
	Penicillin	4	4%
	Ciprofloxacin	3	3%
	Piperacillin	3	3%
	Tazocin	3	3%
	Cefepime	2	2%
	Ceftazidime	2	2%
	Imipenem	2	2%
	Meropenem	2	2%
	Ceftriaxone	1	1%
	Sulfonamide	1	1%
	Colistin	1	1%
	Erythromycin	1	1%

According to Table 3, empirical antibiotics were initiated in 80 (81%) patients. Proper antibiotic coverage was initiated within a week for 53 (53%) patients. Eight (8%) patients had proper antibiotic coverage after one to three months, while 46 (46%) patients received antibiotic treatment for six weeks.

**Table 3:** Characteristics of the antibiotic treatment given to the patients diagnosed with infective endocarditis.

Variables		n N=99	%
Were empirical antibiotics initiated at the ED presentation?		80	81%
Time till proper antibiotic coverage was initiated according to the guidelines	Within one day	26	26%
	Within three days	17	17%
	More than a week	14	14%
	Within a week	10	10%
	Within a month	3	3%
	Within two months	3	3%
	Three months or more	2	2%
Duration of correct antibiotic	Within six weeks	46	46%
	More than seven weeks	12	12%
	Within four weeks	8	8%
	Within one week	5	5%
	Within two weeks	4	4%
	Within five weeks	4	4%
	Within three weeks	1	1%

The echocardiography findings are shown in Table 4. Our study found that vegetation was present in a single valve in more than half of the patients (n = 61, 62%). The mitral valve had the highest incidence of vegetation (n = 24, 24%), followed by the aortic valve (n = 21, 21%). Moreover, follow-up echocardiography was done in 52 patients (52%), out of whom 43 patients (43%) had regressed vegetation while 9 patients (9%) had no vegetation regression.

**Table 4:** The echocardiography findings of the patients diagnosed with infective endocarditis.

Variables		n (N=99)	%
Involvement of single or multiple valves visualized by echocardiography	Single	61	62%
	Multiple	11	11%
Location of vegetation by echocardiography:	Mitral valve	24	24%
	Aortic valve	21	21%
	Right heart	12	12%
	Tricuspid valve	8	8%
	Mitral valve and aortic valve	6	6%
	Mitral and tricuspid	3	3%
	Pulmonary valve	2	2%
	Conduit	2	2%
	Mitral valve and right heart	1	1%
	Tricuspid valve and right heart	1	1%
	Pulmonary valve and right heart	1	1%
	On Atrium	1	1%
	Prosthetic valve	1	1%
Resolution of the vegetation after treatment on follow-up echocardiography	Regressed	43	43%
	Did not regressed	9	9%

Table 5 summarizes the patient’s outcomes. The patients with valve repair were 25 (25%), and the patients requiring ICU admission were 47. Only six (6%) patients received outpatient treatment. Most patients were hospitalized for at least seven days (n = 62, 62%), and 18 (18%) patients had died due to infective endocarditis.

**Table 5:** Infective endocarditis patients’ outcomes regarding their surgery, hospital length of stay, outpatient treatment, and survival.

Variables		n (N=99)	%
Patients that required valve replacement		25	25%
Patients requiring ICU admission		47	47%
Hospital length of stay	7–49 days	62	63%
	50–182 days	19	19%
	7 days	9	9%
	182–365 days	1	1%
Outpatient treatment only		6	6%
Survival to hospital discharge		81	82%

## Discussion

Our study is the first to be done in Saudi Arabia on the quality of care given to infective endocarditis patients. We managed to collect a substantial number of patients despite this being an uncommon disease.

The group with the highest incidence we found in our study was the group aged 41-65 years. This is similar to studies done in Hong Kong and Turkey [5,9]. In contrast to studies done in Pakistan, where the mean age was 29 years, India and Morocco reported a mean age of 28 years [10-12]. These changes in age distribution could be attributed to the decreasing incidence of rheumatic heart disease [13]. In our study, we found the most common chief complaints were fever, shortness of breath, and fatigue, which was similar to a retrospective study done in 25 countries that reports fever as the most common symptom [14].

In our study, we observed that the most common organism reported was Staphylococcus aureus (18%). A large international observational study also reports that *S. aureus* is the most common organism in native valve endocarditis [13]. Similarly, in another study that was done in Saudi Arabia, *S. aureus* was also reported as the most common followed [3]. This is in contrast with a study done in Oman that found that Streptococcus was the most common organism [15]. Streptococcus spp. was also the most common organism reported in a study in Lebanon [16]. Moreover, in South American regions, it was found that 26% of infective endocarditis cases are caused by Streptococci spp. [17]. This could be attributed to the disparity of risk factors, such as intravenous drugs and rheumatic heart disease, or be time-related since our study was done in a later period [17].

Empirical antibiotics were initiated in 80 patients in our study, which is the recommended management by the European Society for Cardiology [18]. Moreover, the ESC recommends antibiotic treatment for at least six weeks, which is what 46% of the patients in the study had received [18].

Half of the patients in our study had vegetation in a single valve, which is in accordance with a study done in South Korea [19]. The two most commonly affected valves in our study were the mitral valve and the aortic valve. This result is in agreement with the findings from the International Collaboration on Endocarditis, where the mitral valve had the highest incidence, approximately 40%, followed by the aortic valve [13]. Also, in another study done in Saudi Arabia, similar findings were reported [3]. Moreover, in our study, we report regression of vegetation in 43 patients out of 53 patients who have had follow-up echocardiography. A study reports that different antibiotic treatments are associated with a different reduction in vegetation size; for example, a 45% reduction in vegetation size is reported to be associated with vancomycin-related treatment [20].

Surgical valve treatment was required in 25% of the patients included in our study. This number is lower than what was reported in the International Collaboration on Endocarditis, which was 48.2% [13]. We found that the mortality rate in our study was lower when compared to international studies that mentioned mortality occurring in 30-40% of cases [21,22].

Study limitations

This study is limited due to it being a retrospective study in design. Moreover, it is conducted in a single teaching hospital rather than in multiple centers. Another limitation is that, compared to other international registries, our study had a low number of patients. This study shows that developing a national registry would be beneficial for the treatment and outcomes of infective endocarditis.

## Conclusions

Infective endocarditis is a rare disease with high mortality and morbidity. In addition, the decreased incidence of rheumatic heart disease has shifted infective endocarditis to a disease that affects mostly older patients. This change also resulted in a difference in microbiology, with *S. aureus* being the most common causative organism.
